# RACK1 and RPS6 as independent prognostic biomarkers in oral squamous cell carcinoma: a five-year survival analysis

**DOI:** 10.3389/froh.2026.1819788

**Published:** 2026-04-14

**Authors:** Sunita Gupta, Chetna Chaudhary, Shikha Gupta, Vineeta Batra, Anshuman Kumar, Hema Malini Iyer, Sujoy Ghosh, Bidhan Chandra Koner, Ritika Shrivastav

**Affiliations:** 1 Department of Oral Medicine and Radiology, Maulana Azad Institute of Dental Sciences, University of Delhi, New Delhi, India; 2 Department of Pathology, Govind Ballabh Pant Hospital, New Delhi, India; 3 Department of Surgical Oncology, Dharamshila Narayana Superspeciality Hospital, Delhi, India; 4 Department of Pathology, Dharamshila Narayana Superspeciality Hospital, Delhi, India; 5 Department of Biochemistry, Maulana Azad Medical College, New Delhi, India

**Keywords:** biomarkers, immunohistochemistry, oral squamous cell carcinoma, prognosis, RACK1, RPS6, survival

## Abstract

**Background:**

This study aimed to investigate the Clinicopathologic & prognostic significance of Receptor for Activated C Kinase 1 (RACK1) and Ribosomal Protein S6 (RPS6) in Oral Squamous Cell Carcinoma (OSCC) through immunohistochemistry.

**Methods:**

Formalin-fixed, paraffin-embedded tumor tissues of 100 OSCC cases who were treated primarily via surgery were retrieved and immunohistochemical analysis was done for RACK1 & RPS6. Clinicopathological data was gathered for these subjects and each was followed up for a period of 5 years from initial diagnosis. Association of expression of marker with clinicopathological parameters was done using Chi-square test. Prognostic significance was evaluated with Cox proportional hazards regression, Kaplan–Meier method, and receiver operating characteristic (ROC) analyses.

**Results:**

High expression of RACK1 and RPS6 was significantly associated with age (*p* = .005, *p* = .04), nodal involvement (*p* = .01, *p* = .05), histopathological grading (*p* = <0.001), treatment (*p* = 0.04, *p* = 0.02), and recurrence (*p* = .001, *p* = .03). Subjects with high expression of RACK1 & RPS6 were significantly associated with reduced overall (OS), disease-free (DFS), and cancer-specific survival (CSS) (*p* < 0.001). Co-expression of both markers correlated with the worst prognosis in terms of OS, DFS and CSS (*p* < 0.001). Multivariate Cox Regression analysis showed that both the markers can serve as independent prognostic marker in OSCC. Strong positive linear correlation (R^2^ = 0.718) was observed between expression of RACK1 & RPS6. ROC analysis demonstrated high sensitivity and specificity of both the markers in predicting OS, DFS and CSS.

**Conclusion:**

The results of the present study suggest that RACK1 & RPS6 are associated with tumor progression and invasion in OSCC. Individual as well as combined expression of both the markers may be used as prognostic biomarker in OSCC for patient stratification and personalized treatment planning.

## 1 Introduction

Oral Squamous cell carcinoma (OSCC) is the most common malignancy of the oral cavity accounting for nearly 90% of all oral cancers (1). Annually, approximately 77,000 new cases of OSCC are diagnosed in India, which is about 1/4th of the global estimates (2). The rising number of cases in India is attributable to an increase in smokeless tobacco consumption among younger individuals. Despite several advances in the diagnosis and treatment, the survival rate is approximately 50% in India in comparison to about 68% in developed countries (3). The low survival rate is attributable to late diagnosis, locoregional recurrence and lack of reliable molecular markers. Therefore, there is a need to identify molecular markers that can aid in patient stratification and individualized treatment planning in order to improve prognosis.

Receptor for activated C Kinase 1 (RACK1) is a highly conserved adaptor protein found to regulate scaffolding of key signalling proteins such as cell migration and adhesion through interaction between receptors on cell surface and cytoskeleton elements (4, 5). It has been reported that 3 different pathways are involved in RACK1 regulation in cancer cells- src kinase for cell migration, Focal adhesion kinase (FAK) for cellular adhesion and Epithelial mesenchymal transition for promoting migration and invasion of cancer cells (6–8). RACK1 has been shown to be implicated in tumor invasion and metastasis in numerous cancers including breast cancer (9), oesophageal cancer (10), colon cancer (11), and non-small cell lung cancer (12); however, its role in OSCC is poorly elucidated with lack of studies on its clinical and prognostic implication.

Ribosomal protein S6 (RPS6), a component of 40S ribosomal unit, is one of the only 2 ribosomal proteins known to be phosphorylated (13). It plays an important role in translation of protein, cell growth, DNA repair, apoptosis as well as in tumor development and invasion (14). It is a downstream regulator of mammalian target of rapamycin complex 1 (mTORC1), which is known to be implicated in many cancers, including OSCC (15). Overexpression of RPS6 has been reported in various tumours including non-small cell lung cancer (16), breast cancer (17), renal cell carcinoma (18), and ovarian cancer (19), and the expression was also correlated with histopathologic grade in these cancers**.** RPS6 has been shown to increase cell proliferation by downregulating p16 and p21 and cause invasion of tumor by upregulation of Vimentin, matrix metalloproteinases, and N-Cadherin (16, 20). However, limited evidence exists on clinicopathologic role of RPS6 in OSCC.

Therefore, the aim of the present study was to evaluate the immunohistochemical expression of RACK1 & RPS6 and correlate the same with clinicopathological parameters including prognosis in subjects with OSCC. This study is unique in simultaneous evaluation of expression of RACK1 & RPS6, both of which represent 2 distinct levels of cellular regulation, namely scaffolding and protein synthesis. Furthermore, our study addresses a significant gap in the current literature, as there is a paucity of OSCC-specific data on these markers, especially in the context of survival outcomes.

## 2 Materials and methods

### 2.1 Subject selection

This study was undertaken in the Department of Oral Medicine & Radiology, Maulana Azad Institute of Dental Sciences (MAIDS), New Delhi in collaboration with Department of Pathology, GB Pant Institute of Postgraduate Medical Education and Research (GIPMER), New Delhi and Department of Surgical Oncology and Department of Pathology at Dharamshila Narayana Superspeciality Hospital (DNSH), New Delhi. Formalin fixed, paraffin embedded (FFPE) tissue specimens from OSCC who underwent surgery as the primary treatment modality with/without chemotherapy and/or radiotherapy between 2017 & 2019 were retrieved from the archives of Department of Pathology at DNSH. Prior informed consent was obtained from all the patients for use of their clinicopathological data and tissue for the research purpose. The study was approved by the Institutional Ethical Committee.

Clinical records including age, sex, site of tumor, tumor size, lymph node involvement, imaging data, and clinical TNM staging according to the American Joint Committee on Cancer (AJCC), 8th edition were obtained from the Department of Surgical Oncology at DNSH. Hematoxylin and eosin (H&E)-stained slides were reviewed, and histopathological features—such as depth and pattern of invasion, lymphovascular invasion (LVI), perineural invasion (PNI), and pathological TNM staging—were independently assessed by two experienced pathologists. All patients were followed up at MAIDS for a minimum duration of five years, during which Overall Survival (OS), Disease-Free Survival (DFS), and Cancer-Specific Survival (CSS) were recorded.

### 2.2 Immunohistochemistry (IHC)

IHC was performed at the Department of Pathology, GIPMER. FFPE blocks were sectioned at 4 μm thickness onto poly-L-lysine coated slides and subjected to immunostaining using the following primary antibodies:

- RACK1 (Rabbit polyclonal antibody; dilution 1:50; Catalogue No. PA5-87361, Thermo Fisher/Invitrogen)
- RPS6 (Ser235/236) (Mouse monoclonal antibody; dilution 1:75; Catalogue No. MA5-15140, Thermo Fisher/Invitrogen)

All IHC steps were performed according to the manufacturer's protocol. The slides were independently evaluated by two expert pathologists. Any interobserver variability was resolved through discussion to reach a consensus score. For each case, five randomly selected high-power fields (40×) at the tumor invasive front were analysed using a light microscope (Nikon, NIS Elements software version 5.30). A formalin-fixed, paraffin-embedded section of colon carcinoma tissue was used as a positive control.

### 2.3 Scoring

**Figure 1:**
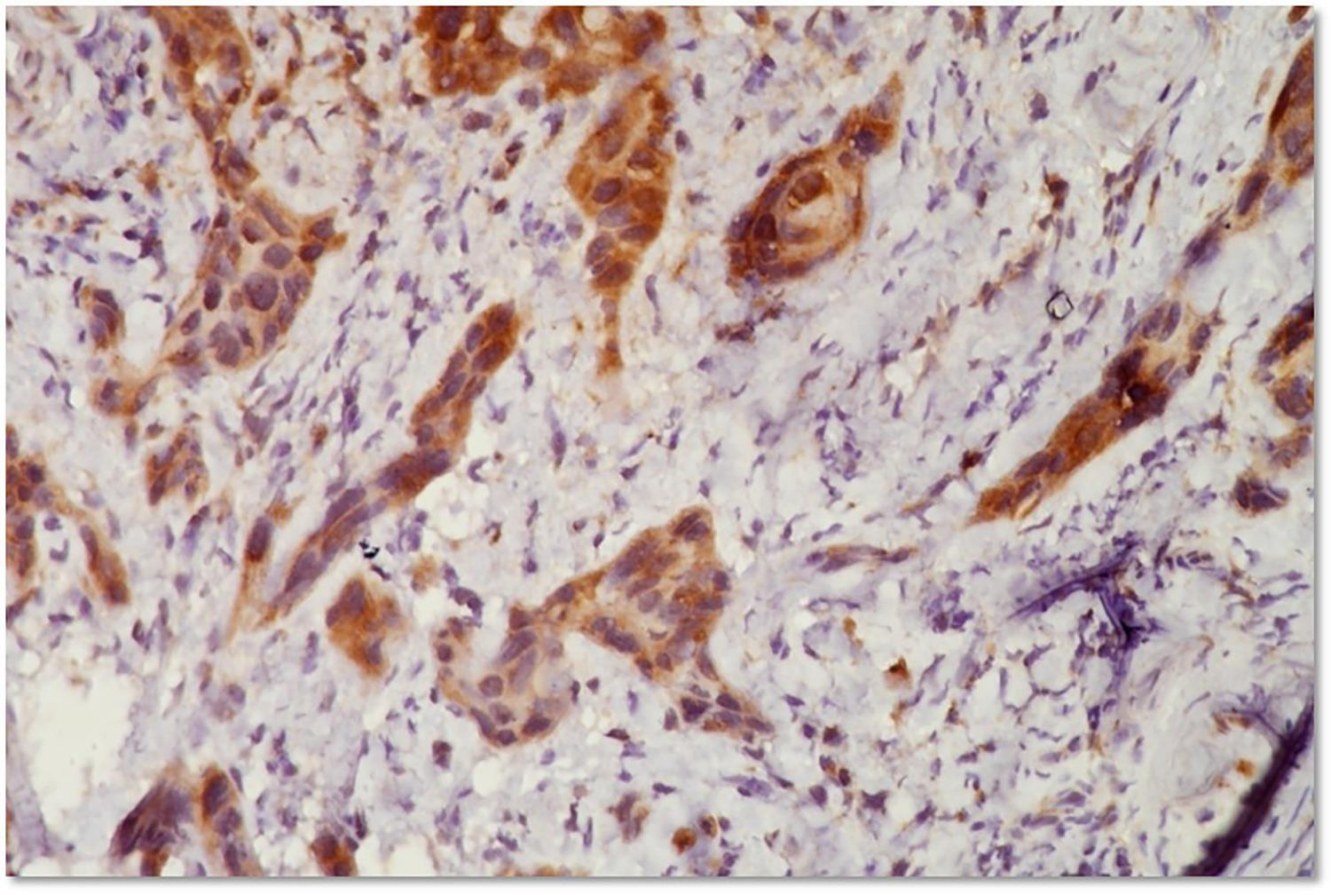
Immunohistochemical staining for RACK1 in a section of moderately differentiated OSCC. Positive staining is indicated by brown colour predominantly in the cytoplasm of malignant epithelial cells. The surrounding stromal tissue shows no immunoreactivity. Magnification: 400x.

For RACK 1, staining was evaluated based on two key parameters: the percentage of positively stained cells and the intensity of staining. The extent of staining is graded on a scale from 0 to 4. Grade 0 indicates a negative result with no stained cells, Grade 1 represents weak staining with 1%–25% of cells showing positivity, Grade 2 corresponds to moderate staining observed in 26%–50% of cells, Grade 3 indicates strong staining in 51%–75% of cells, and Grade 4 reflects very strong staining in 76%–100% of cells. In parallel, the staining intensity is evaluated on a scale from 0 to 3, where Grade 0 denotes no detectable staining, Grade 1 is characterized by light yellow staining, Grade 2 shows deep yellow coloration, and Grade 3 is marked by brown staining (Figure 1). The final IHC score is derived by multiplying the percentage score with the intensity score. Based on this product, a total score of 0–6 is categorized as low expression, whereas a score greater than 6 is considered as high expression.

**Figure 2:**
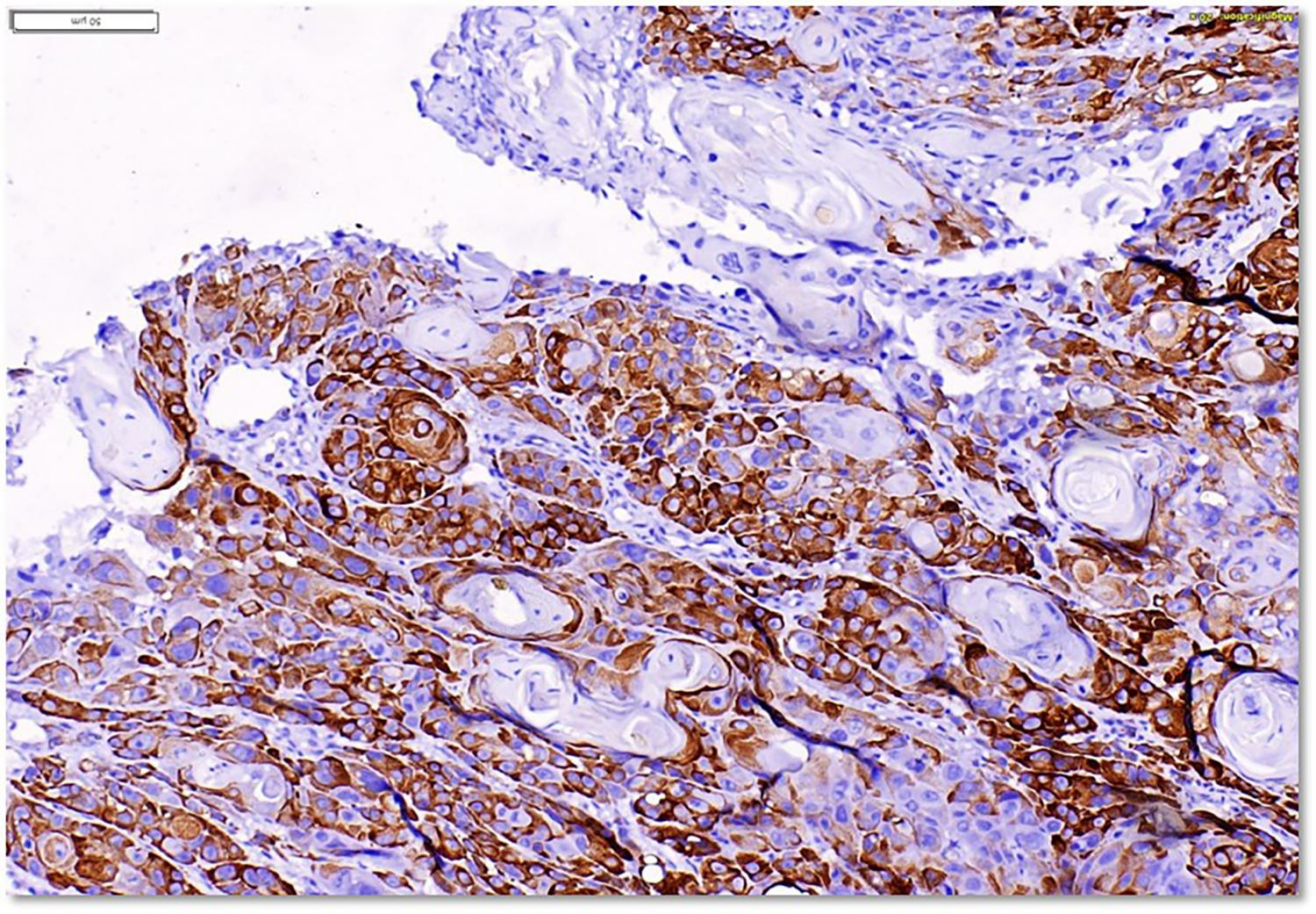
Immunohistochemical staining of RPS6 in section of poorly differentiated OSCC. Tumor islands show strong membranous and partial cytoplasmic staining (brown). Areas of keratin pearl formation are also visible within the tumor nests. Magnification: 200x.

For RPS6, The grading of staining was based on the percentage of positive tumor cells and was categorized as follows: Grade 0 is less than 1% of tumor cells positive, Grade 1 corresponds to 1%–25% positivity, Grade 2 indicates 26%–50% of tumor cells showing positive staining, Grade 3 reflects 51%–75% positivity (Figure 2), and Grade 4 represents 76%–100% of tumor cells being positively stained. Grade 0 & 1 was considered low expression and Grade 2, 3 and 4 as High expression.

### 2.4 Statistical analysis

Data was compiled using Microsoft Excel (v2019) and analysed with SPSS software (version 21, IBM). Categorical variables were presented as frequencies and percentages, while continuous variables were reported as mean values with standard deviations (SD). The relationship between RACK1 and RPS6 expression and various clinicopathological parameters was assessed using the Chi-square test. Kaplan–Meier survival analysis was performed to evaluate OS, DFS, and CSS, and comparisons were made using the Independent Samples t-test. Cox regression analysis was employed to determine hazard ratios for survival outcomes after adjusting for confounding variables. To assess the correlation between RACK1 and RPS6 expression, Spearman's correlation coefficient was used. Receiver Operating Characteristic (ROC) curves and Area Under the Curve (AUC) were generated to evaluate the sensitivity and specificity of RACK1 and RPS6 expression in predicting OS, DFS, and CSS. *P*-value ≤ 0.05 was considered statistically significant.

## 3 Results

### 3.1 Sociodemographic characteristics of study population

As seen in Table 1, a total of 100 histopathologically confirmed OSCC patients were included in the study, with a male predominance (94%) and majority patients were under 60 years of age (78%). The most common site of involvement was buccal mucosa followed by tongue. Majority subjects (86%) belonged to advanced stage. Histopathologically, most tumours were moderately differentiated (60%), with 86% showing less invasive depth. In majority of the cases, lymphovascular invasion and peripheral invasion was absent. Combined surgery with radiotherapy and chemotherapy was the most common treatment modality (84%). Only 47% of the subjects survived at the end of study period.

**Table 1:** Clinico-pathologic characteristics of study population (*N* = 100).

Characteristics	Subgroup	*n* (%)
Age	<60 years	78 (78%)
	>60 years	22 (22%)
Gender	Male	94 (94.00%)
	Female	6 (6.00%)
Site	Buccal Mucosa	41 (47.00%)
	Tongue	28 (28.00%)
	Floor of the mouth	0 (0)
	Lip	2 (2.00%)
	Others	29 (29.00%)
Tumor size (T)	T0 + T1	14 (14.00%)
	T3 + T4	86 (86.00%)
Nodal status (N)	N0 + N1	67 (67%)
	N2 + N3	33 (33%)
Metastasis (M)	Present	6 (6%)
	Absent	94 (94%)
Histopathological Grade	Well-differentiated	36 (36%)
	Moderately differentiated	60 (60%)
	Poorly differentiated OSCC	4 (4.00%)
Histological depth	Less invasive	86 (86%)
	Moderately invasive	10 (10%)
	Deep invasive	4 (4%)
Histological margin	Less than 1 mm	45 (45%)
	1–5 mm	39 (39%)
	More than 5 mm	16 (16%)
Lymph node status	Positive	46 (46%)
	Negative	54 (54%)
Extracapsular spread	Present	30 (30.00%)
	Absent	70 (70.00%)
Lymphovascular invasion	Present	6 (6.00%)
	Absent	94 (94.00%)
Peripheral Invasion	Present	14 (14.00%)
	Absent	86 (86.00%)
Treatment	Surgery	0.00%
	Surgery + RT	16 (16%)
	Surgery +CT	0.00%
	Surgery + RT + CT	84 (84%)
Recurrence	Present	16 (16%)
	Absent	84 (84%)
Survival status	Survived	47 (47%)
	Not survived	53 (53%)

### 3.2 Association of RACK1 and RPS6 expression with clinicopathological parameters in OSCC

We correlated the immunohistochemical expression of RACK1 & RPS6 with clinico-pathological parameters in OSCC and the findings are summarised in Table 2. Positive expression of RACK1 & RPS6 was seen as brown cytoplasmic staining of tumor cells in OSCC as seen in Figures 1, 2. For RACK1, low expression was seen in 34% subjects while 66% showed high expression. Similarly, Grade 1 & 2 staining of RPS6 was seen in 38% subjects while 62% showed grade 3 & 4 staining.

**Table 2:** Association of RACK-1 and RPS6 expression with clinicopathological parameters in patients with oral squamous cell carcinoma (OSCC).

Clinicopathologic Parameter	Subgroups	RACK-1		*p*-value	RPS6		*p*-value
		Low	High		Low	High	
Age	< 60 years	33 (42.3)	45 (57.7)	**0** **.** 005*	25 (32.4)	53 (68.84)	**0** **.** 04*
	>60 years	1 (4.55)	21 (95.45)		2 (9.1)	20 (90.9)	
Gender	Male	33 (35.1)	61 (64.9)	0.33	26 (27.7)	68 (72.3)	0.48
	Female	1 (16.7)	5 (83.3)		1 (16.7)	5 (83.3)	
Site	Buccal Mucosa	17 (41.5)	24 (58.5)	0.48	14 (34.1)	27 (65.9)	0.35
	Tongue	12 (42.9)	16 (57.1)		8 (28.6)	20 (71.4)	
	Floor of the mouth	0 (0)	0 (0)		0 (0)	0 (0)	
	Lip	0 (0)	2 (100)		0 (0)	2 (100)	
	Others	5 (17.2)	24 (82.8)		5 (17.2)	24 (82.8)	
T (Tumor size)	T0 + T1	8 (57.1)	6 (42.9)	**0** **.** 05*	6 (42.9)	8 (57.1)	0.13
	T3 + T4	26 (30.2)	60 (69.8)		21 (24.4)	65 (75.6)	
N (Nodal involvement)	N0 + N1	29 (38.9)	38 (61.1)	**0** **.** 01*	23 (31.5)	44 (68.5)	**0** **.** 05*
	N3 + N4	5 (17.4)	28 (82.6)		4 (17.4)	29 (82.6)	
M (Metastases)	Absent	34 (36.2)	60 (63.8)	0.07	27 (28.7)	67 (71.3)	0.14
	Present	0 (0)	6 (100)		0 (0)	6 (100)	
Histopathological grading	Well-differentiated	21 (58.3)	15 (41.7)	**<0** **.** 001*	19 (52.8)	17 (47.2)	**<0** **.** 001*
	Moderately differentiated	13 (21.7)	47 (78.3)		8 (13.3)	52 (86.7)	
	Poorly differentiated OSCC	0 (0)	4 (100)		0 (0)	4 (100)	
Depth	Less invasive	29 (33.7)	57 (66.3)	0.20	24 (27.9)	62 (72.1)	0.45
	Moderately invasive	5 (50)	5 (50)		3 (30)	7 (70)	
	Deep invasive	0 (0)	4 (100)		0 (0)	4 (100)	
Margin	Less than 1 mm	18 (40)	27 (60)	0.36	16 (35.6)	29 (64.4)	0.10
	1–5 mm	10 (25.6)	29 (74.4)		6 (15.4)	33 (84.6)	
	More than 5 mm	6 (37.5)	10 (62.5)		5 (31.3)	11 (68.8)	
Lymph node status	Positive	21 (38.9)	33 (61.1)	0.18	17 (31.5)	37 (68.5)	0.19
	Negative	13 (28.3)	33 (71.7)		10 (21.7)	36 (78.3)	
Extracapsular spread	Absent	26 (37.1)	44 (62.9)	0.64	20 (28.6)	50 (71.4)	0.39
	Present	8 (26.7)	22 (73.3)		7 (23.3)	23 (76.7)	
Lymphovascular invasion	Absent	33 (35.1)	61 (64.9)	0.33	26 (27.7)	68 (72.3)	0.48
	Present	1 (16.7)	5 (83.3)		1 (16.7)	5 (83.3)	
Peripheral Invasion	Absent	28 (32.6)	58 (67.4)	0.32	21 (24.4)	65 (75.6)	0.13
	Present	6 (42.9)	8 (57.1)		6 (42.9)	8 (57.1)	
Treatment	Surgery + RT	9 (56.3)	7 (43.8)		8 (50)	8 (50)	**0** **.** 04*
	Surgery + CT	25 (29.8)	59 (70.2)		19 (22.6)	65 (77.4)	
Recurrence	Absent	33 (39.3)	51 (60.7)	**0** **.** 001*	26 (31)	58 (69)	**0** **.** 03*
	Present	1 (6.3)	15 (93.8)		1 (6.3)	15 (93.8)	
Survival	Not Survived	1 (1.9)	52 (98.1)	**0** **.** 001*	0 (0)	53 (100)	**<0** **.** 001*
	Survived	33 (70.2)	14 (29.8)		27 (57.4)	20 (42.6)	

Chi-square test; *p* value < 0.05 is considered statistically significant.

Values highlighted in bold and * indicate statistically significant.

RACK1 expression was significantly associated with age, T stage, N stage, histopathologic grade, treatment, recurrence, and the survival status. RPS6 showed significant correlation with age, N stage, histopathologic grade, treatment, recurrence, and the survival status. Subjects more than 60 years, Advanced T stage and N stage showed high expression of RACK1 & RPS6. All poorly differentiated and majority moderately differentiated tumours showed high expression. Nearly all patients who did not survive and majority of those who underwent chemotherapy after surgery showed high expression of RACK1 & RPS6.

### 3.3 Survival analysis based on expression of RACK1 & RPS6

**Figure 3:**
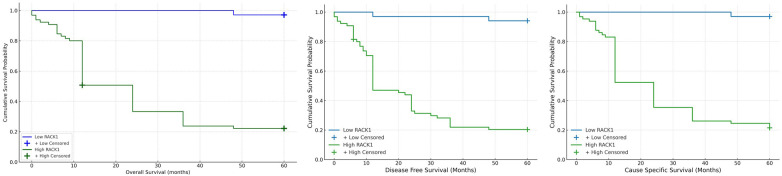
Kaplan–meier survival analysis based on RACK1 expression in OSCC patients kaplan–meier plots show the association between RACK1 expression levels and overall survival (OS), disease-free survival (DFS), and cancer-specific survival (CSS) in patients with OSCC. Patients with low RACK1 expression (blue line) had significantly better survival outcomes compared to those with high RACK1 expression (green line). Censored cases are indicated with “+” markers. Differences between groups were statistically significant (*p* < 0.001, log-rank test for all).

**Figure 4:**
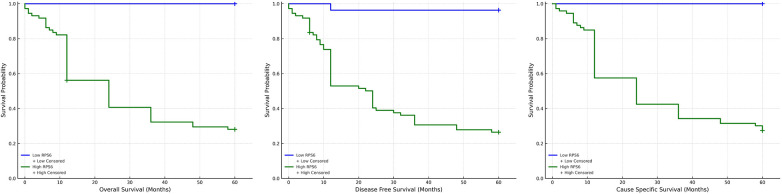
Kaplan–meier survival analysis based on RPS6 expression in OSCC patients kaplan–meier plots depict survival outcomes stratified by RPS6 expression levels for overall survival (OS), disease-free survival (DFS), and cancer-specific survival (CSS) in OSCC patients. Low RPS6 expression (blue line) was significantly associated with improved survival across all endpoints, whereas high RPS6 expression (green line) correlated with worse prognosis. Censored cases are indicated by “+”. Statistical differences were significant (*p* < 0.001, log-rank test for all).

We examined the individual and combined expression of RACK1 & RPS6 with Overall Survival (OS), Disease-free Survival (DFS) and Cancer-specific survival (CSS). It was observed that subjects with high expression of RACK1 & RPS6 exhibited significantly low OS, DFS & CSS as compared to those with low expression (Table 3) (Figures 3, 4). Subjects with low expression of both the markers showed significantly higher survival compared to those with high expression of both. The 5-year overall survival (OS) rate was significantly higher in the low RACK1 expression cohort, reaching 97.05%, compared to only 22.72% in the high RACK1 expression group. Similarly, patients with low RPS6 expression demonstrated a 5-year OS of 100%, while those with high RPS6 expression had a markedly reduced OS of 28.76%. In terms of disease-free survival (DFS), the low RACK1 expression group exhibited a 5-year DFS of 94.11%, whereas the high RACK1 expression group showed a significantly lower DFS of 21.21%. For RPS6, patients with low expression had a 5-year DFS of 96.29%, in contrast to just 27.39% in those with high expression levels. For CSS, Low RACK1 expression group showed a 5-year CSS of 33% in contrast to 6% in high expression group. Whereas, those with low RPS6 had a 5-year survival rate of 21% compared to 27% for high expression group. The mean OS, DFS & CSS was significantly more in Non-recurrence group as compared to those with recurrence.

**Table 3:** Association of RACK1 and RPS6 expression with overall survival (OS), disease-free survival (DFS), and cancer-specific survival (CSS) in patients with oral squamous cell carcinoma (OSCC).

Marker	Expression	OS (Mean ± SD, Months)	DFS (Mean ± SD, Months)	CSS (Mean ± SD, Months)
RACK1	Low (*n*=34)	59.65 ± 2.05	58.24 ± 8.42	59.65 ± 2.05
	High (*n*=66)	26.09 ± 20.93	24.52 ± 20.91	27.91 ± 21.19
	*p* value	**<0** **.** 001*	**<0** **.** 001*	**<0** **.** 001*
RPS6	Low (*n*=27)	60 ± 0	58.22 ± 9.23	60 ± 0
	High (*n*=73)	29.18 ± 22.10	27.75 ± 22.28	30.82 ± 22.10
	*p* value	**<0** **.** 001*	**<0** **.** 001*	**<0** **.** 001*
Combined RACK1 & RPS6	Both Low (*n*=28)	59.65 ± 2.05	58.24 ± 8.42	59.65 ± 2.05
	Both High (*n*=66)	26.09 ± 20.9	24.52 ± 20.9	27.91 ± 21.1
	*p* value	**<0** **.** 001*	**<0** **.** 001*	**<0** **.** 001*
Recurrence Status	Recurrence	25.5 ± 16.32	15.93 ± 10.01	25.5 ± 16.32
	Non-Recurrence	39.75 ± 23.83	39.79 ± 23.82	41.21 ± 23.19
	*p* value	**<0** **.** 001*	**<0** **.** 001*	**<0** **.** 001*

Independent sample t-test, *p* value <0.05 is considered statistically significant.

Values highlighted and bold and * indicate statistically significant.

### 3.4 Multivariate Cox regression analysis of RACK1 & RPS6 with OS, DFS & CSS

A series of Cox regression models (Models 1a–4d) were used to evaluate the prognostic significance of RACK1 and RPS6 expression in relation to Overall Survival (OS), Disease-Free Survival (DFS), and Cancer-Specific Survival (CSS). Model 1a depicts patients with high RACK1 and RPS6 have significantly higher risk of death than those with low expression even after adjusting for various clinicopathological factors such as age, gender, lymph node, tumor stage, histopathological grade, metastases, TNM stage and treatment as per Model 2b & Model 3c. High RACK1 and RPS6 remain strong independent predictor of poor survival in OSCC**.** Model 4d shows fully adjusted model where high RACK1 and RPS6 expression is associated with higher risk of death, confirming it as a strong, independent prognostic marker (Table 4)**.**

**Table 4:** Multivariate Cox proportional hazards regression models of RACK1 and RPS6 expression with OS, DFS & CSS in OSCC.

Survival Outcome	Marker	Model 1^a^	Model 2^b^	Model 3^c^	Model 4^d^
		Hazard Ratio (*p* value)	Hazard Ratio (*p* value)	Hazard Ratio (*p* value)	Hazard Ratio (*p* value)
Overall Survival	RACK1 (High/Low)	3.21 (0.001*)	3.16 (0.001*)	3.07 (0.001*)	2.26 (0.001*)
	RPS-6 (High/Low)	6.21 (0.001*)	5.08 (0.001*)	4.86 (0.001*)	4.64 (0.001*)
Disease Free Survival	RACK1 (High/Low)	4.78 (<0.001*)	4.2 (<0.001*)	2.99 (0.001*)	2.8 (<0.001*)
	RPS-6 (High/Low)	4.67 (<0.001*)	3.97 (<0.001*)	3.18 (<0.001*)	2.64 (<0.001*)
Cause Specific Survival	RACK1 (High/Low)	3.74 (0.001*)	2.93 (0.001*)	2.53 (0.001*)	1.80 (0.01*)
	RPS-6 (High/Low)	4.69 (0.001*)	2.64 (0.001*)	2.53 (0.001*)	2.16 (0.01*)

^a^ Unadjusted.

^b^ Adjusted for confounders age and gender.

^c^ Adjusted for lymph node, histopathological grade, TNM staging, metastases, treatment.

^d^ Adjusted for all the covariates in b and c model.

*P* value < 0.05 is considered statistically significant (marked with *).

### 3.5 Correlation between RACK1 & RPS6 expression & ROC curve analysis

**Figure 5:**
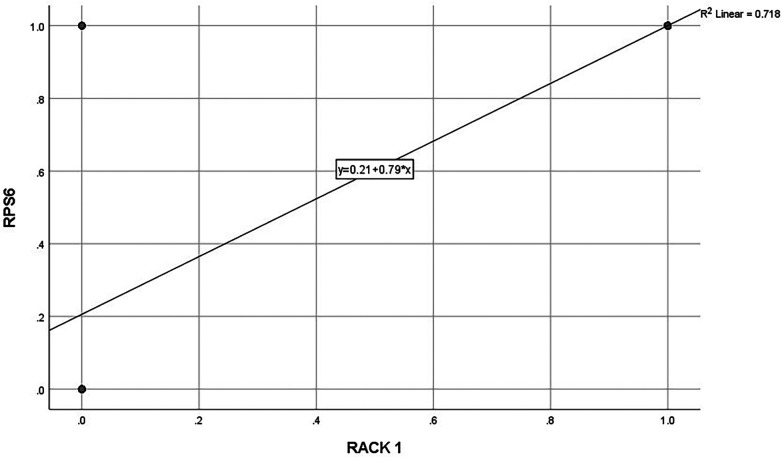
Linear regression analysis showing the relationship between RACK1 and RPS6 expression levels. A positive linear correlation was observed between RACK1 and RPS6, as represented by the regression equation y = 0.21 + 0.79xy = 0.21 + 0.79xy = 0.21 + 0.79x and coefficient of determination R2 = 0.718R^2 = 0.718R2 = 0.718. The data points are plotted with a best-fit line, indicating a moderately strong association between the two variables.

**Figure 6:**
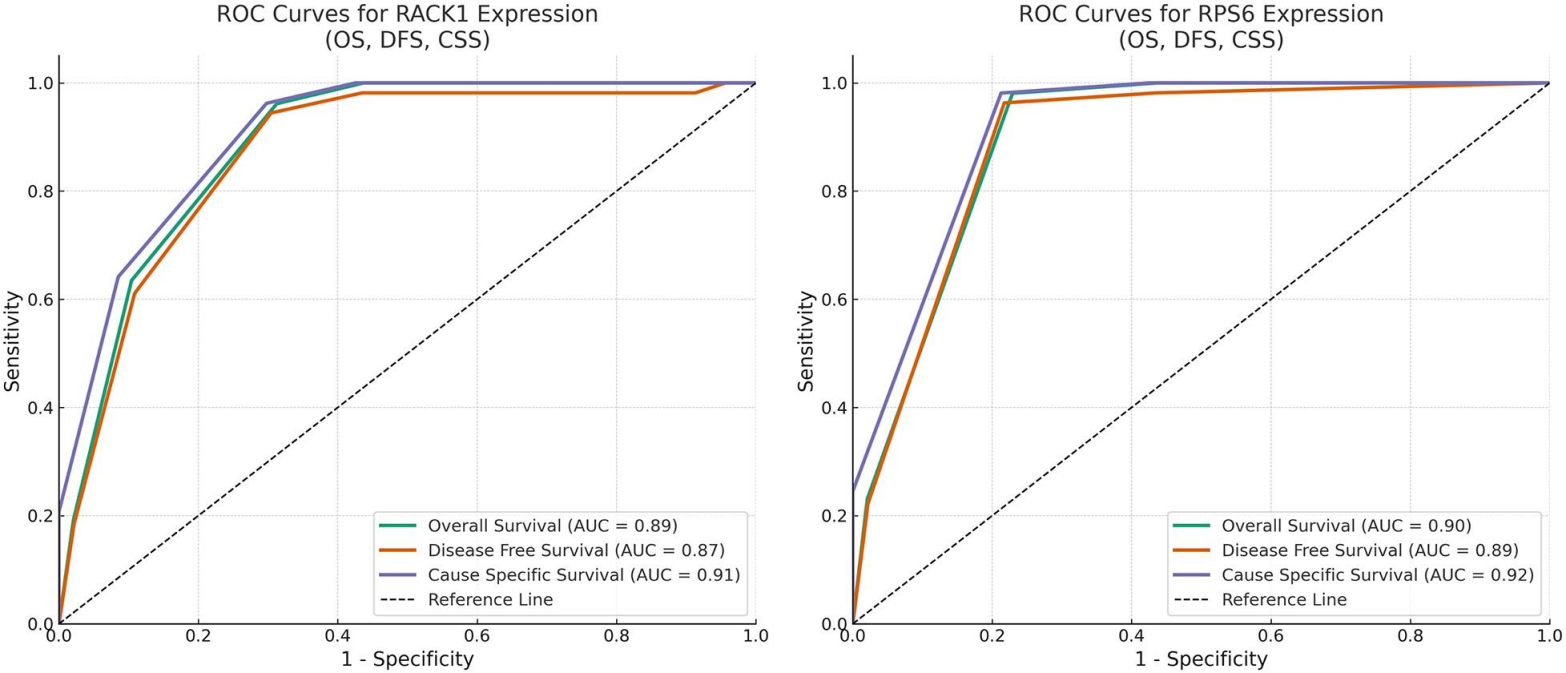
Receiver operating characteristic (ROC) curves for RACK1 and RPS6 expression in predicting survival outcomes. Panels show ROC analyses for RACK1 (left) and RPS6 (right) expression in predicting Overall Survival (OS), Disease-Free Survival (DFS), and Cancer-Specific Survival (CSS). The curves demonstrate strong discriminatory performance across all outcomes, with respective area under the curve (AUC) values of 0.89 (OS), 0.87 (DFS), and 0.91 (CSS) for RACK1, and 0.90 (OS), 0.89 (DFS), and 0.92 (CSS) for RPS6. The dashed diagonal line represents the reference line (AUC = 0.5) indicating no discriminative ability.

A strong positive linear correlation was observed between RACK1 and RPS6 expression levels, as demonstrated by linear regression analysis (R^2^ = 0.718) in Figure 5. The regression equation indicates that higher RACK1 expression is significantly associated with increased RPS6 expression. ROC curve analyses were performed to evaluate the prognostic utility of RACK1 and RPS6 expression levels in predicting Overall Survival (OS), Disease-Free Survival (DFS), and Cancer-Specific Survival (CSS) in OSCC. In Figure 6, it can be seen that both markers exhibited high sensitivity and specificity (AUC- 0.87 to 0.92) in predicting outcomes in terms of OS, DFS & CSS.

## 4 Discussion

The survival rate for OSCC remains low despite the advancements in therapy owing to the lack of reliable diagnostic and prognostic markers. The survival seems to be influenced by several factors, including patient factors, treatment factors, and tumor biological factors (2). Assessment of these factors is crucial to understand how these affect the prognosis, which will enable personalized and targeted therapy thereby improving the prognosis. Over the past few decades, several predictive and prognostic markers have been researched upon but clinical utility is low (3). In the present study, we have evaluated the immunohistochemical expression of RACK1 & RPS6 in OSCC and correlated the expression with clinicopathologic parameters including prognosis.

Our findings demonstrate that high immunohistochemical expression of RACK1 (Receptor for Activated C Kinase 1) is significantly associated with advanced tumor stage, lymph node metastasis, poor histological differentiation, and lower survival outcomes. These results are in line with studies in breast (9), lung (20), and hepatocellular carcinomas (21) where RACK1 has been implicated in promoting tumor proliferation, migration, and invasion via interaction with oncogenic signalling pathways such as Src kinases and PKC (9, 22). RACK1, a scaffolding protein, facilitates signal integration across multiple oncogenic pathways, and its dysregulation can augment epithelial-to-mesenchymal transition (EMT), tumor angiogenesis, and chemoresistance (23, 24). In the context of OSCC, where molecular heterogeneity is significant, RACK1 overexpression may serve as a converging node of multiple oncogenic drivers.

Similarly, RPS6 (Ribosomal Protein S6), a downstream effector of the PI3 K/AKT/mTOR axis, showed robust prognostic relevance. High RPS6 expression correlated with aggressive tumor features and significantly reduced Overall Survival (OS), Disease-Free Survival (DFS), and Cancer-Specific Survival (CSS). RPS6 regulates mRNA translation of pro-proliferative and anti-apoptotic proteins, and its phosphorylation is a hallmark of hyperactive mTOR signalling, commonly observed in OSCC (25, 26). Prior studies in Non-Small Cell Lung Cancer and oesophageal cancers have shown that elevated RPS6 correlates with increased tumor size, invasion, and reduced chemotherapy response (20, 27). Our data align with this paradigm, indicating that RPS6 is not merely a surrogate for mTOR activation but may have independent prognostic and functional implications in OSCC biology.

Notably, this study is among the first to simultaneously evaluate RACK1 and RPS6 in OSCC, providing a dual perspective on two distinct yet interconnected levels of tumor regulation—signal scaffolding and protein synthesis. The observed strong positive correlation (R^2^ = 0.718) between RACK1 and RPS6 expression suggests a functional interplay, potentially via shared regulatory pathways such as mTOR or integrin-linked kinase signalling. Previous reports suggest crosstalk between RACK1 and translational signaling pathways. RACK1 has been shown to promote tumor progression via activation of the AKT/mTOR pathway, while modulation of PI3 K/Akt signaling influences downstream S6 phosphorylation (28). This interplay warrants further mechanistic exploration, particularly to understand whether RACK1 acts upstream or in parallel with RPS6 in OSCC pathogenesis.

From a clinical standpoint, the independent prognostic value of RACK1 and RPS6 across multiple multivariate Cox models—adjusted for age, gender, TNM stage, and differentiation—underscores their potential as biomarkers for risk stratification. Furthermore, ROC analyses demonstrated high sensitivity and specificity for both markers, with RPS6 showing slightly superior predictive performance. Importantly, patients with concurrent low expression of both markers had significantly improved OS, DFS, and CSS, suggesting additive prognostic utility. This dual-marker approach could enhance traditional TNM staging by identifying high-risk individuals who may benefit from more aggressive or targeted therapies, including mTOR inhibitors, Src kinase inhibitors, or novel agents targeting scaffold signalling complexes (29).

This study offers several noteworthy strengths that enhance the validity and clinical relevance of its findings. Firstly, it is the first to simultaneously evaluate the prognostic significance of both RACK1 and RPS6 in OSCC, providing insight into two critical but distinct regulatory axes—signal transduction and protein synthesis. The dual-marker approach enables a more nuanced understanding of tumor progression and allows for more effective risk stratification. Secondly, the study is based on a well-characterized cohort of 100 histopathologically confirmed OSCC patients, with a minimum follow-up of five years, enabling robust analysis of long-term survival outcomes. Third, a comprehensive statistical framework—including multivariate Cox regression, Kaplan–Meier survival analysis, ROC curve evaluation, and correlation modelling—was employed to ensure analytical rigor. The findings are further strengthened by standardized immunohistochemical protocols and dual-pathologist scoring, enhancing reproducibility. Lastly, the inclusion of patients from a high-incidence, underrepresented South Asian population addresses an important regional gap in OSCC biomarker research and supports the translational relevance of these biomarkers in resource-constrained clinical settings. Given the regional heterogeneity in tumor biology and risk factors, biomarker studies rooted in endemic populations are vital for building context-specific precision oncology frameworks (30).

While our findings are compelling, several limitations must be acknowledged. The study's single-institutional design may introduce selection bias. Although we adjusted for multiple confounders, prospective validation in larger, multicentric cohorts is essential. Furthermore, functional studies are needed to elucidate the mechanistic link between RACK1 and RPS6 expression in OSCC progression. RNA-seq, phosphoproteomics, and CRISPR-based loss-of-function studies could provide deeper insight into their regulatory networks. Moreover, while IHC offers practicality in clinical workflows, future studies might explore whether circulating or salivary levels of RACK1 or phosphorylated RPS6 can serve as non-invasive biomarkers, particularly for early detection or post-treatment surveillance.

## Data Availability

The original contributions presented in the study are included in the article/Supplementary Material, further inquiries can be directed to the corresponding author.
